# Carnitine and Depression

**DOI:** 10.3389/fnut.2022.853058

**Published:** 2022-03-14

**Authors:** Ting Liu, Kunhong Deng, Ying Xue, Rui Yang, Rong Yang, Zhicheng Gong, Mimi Tang

**Affiliations:** ^1^Department of Pharmacy, Xiangya Hospital, Central South University, Changsha, China; ^2^National Clinical Research Center for Geriatric Disorders, Xiangya Hospital, Institute for Rational and Safe Medication Practices, Central South University, Changsha, China; ^3^Center of Clinical Pharmacology, The Third Xiangya Hospital, Central South University, Changsha, China

**Keywords:** carnitine, acylcarnitines, depression, bipolar disorder, schizophrenia

## Abstract

Depression has become one of the most common mental diseases in the world, but the understanding of its pathogenesis, diagnosis and treatments remains insufficient. Carnitine is a natural substance that exists in organisms, which can be synthesized *in vivo* or supplemented by intake. Relationships of carnitine with depression, bipolar disorder and other mental diseases have been reported in different studies. Several studies show that the level of acylcarnitines (ACs) changes significantly in patients with depression compared with healthy controls while the supplementation of acetyl-L-carnitine is beneficial to the treatment of depression. In this review, we aimed to clarify the effects of ACs in depressive patients and to explore whether ACs might be the biomarkers for the diagnosis of depression and provide new ideas to treat depression.

## Introduction

Depression is a complex mental disease as a result of sociopsychological, and physiological factors combined. Its main manifestations include disorders of emotion, cognition, behavior, sleep, and appetite (1, 2). The transmission of monoaminergic neurotransmitters and the impairment of neuroplasticity are considered as the core in the pathophysiological mechanism of depression (3).

At present, less than half of the patients with depression are receiving effective treatments, with low cure rate and high recurrence rate (4). Meanwhile, it is still difficult to diagnose depression mainly relying on clinical phenomenology and the judgment of psychiatrists (5). Some studies have found that carnitine supplementation, especially acetyl-L-carnitine (LAC), can significantly improve the symptoms of patients with depression, and is expected to become a new treatment for depression (6). Other clinical studies indicated that the level of LAC in patients with major depressive disorder (MDD) was significantly lower than healthy controls (7). It suggests that carnitine might be involved in the pathological process of depression, which also provides the possibility of carnitine serving as a biomarker for the diagnosis and treatment of depression (7). However, it still remains unknown what types of acylcarnitines (ACs) and their optimal concentrations are helpful for the diagnosis and treatment of depression and other mental illnesses. Systematic studies of this topic are therefore needed for further investigation.

This study mainly reviewed the literature on the determination of ACs in patients with depression or other mental illnesses. It summarized potential ACs as biomarkers of depression and aims to improve our understanding of the carnitine-related pathophysiological mechanism of depression.

## Methods

Studies were searched in the electronic databases: PubMed, Embase, Cochrane Library, and Web of Science. Moreover, we also searched preprint platform to avoid publication bias. The search was performed by two independent reviewers. Medical Subject-Heading (MeSH) and text words were used as search strategy. And the search strategy was modified according to the database type: (“carnitine” OR “acylcarnitine”) AND (“depression” OR “bipolar disorder” OR “schizophrenia” OR “mood disorders”); (“LAC” OR “Acetyl-L-Carnitine”) AND (“antidepressant” OR “antidepression”) and their matching synonyms were used for an adequate search strategy. Additionally, two independent observers extracted data and resolved differences by discussion to maintain the validity and reliability.

## Carnitine and Physiological Function

**Figure 1:**
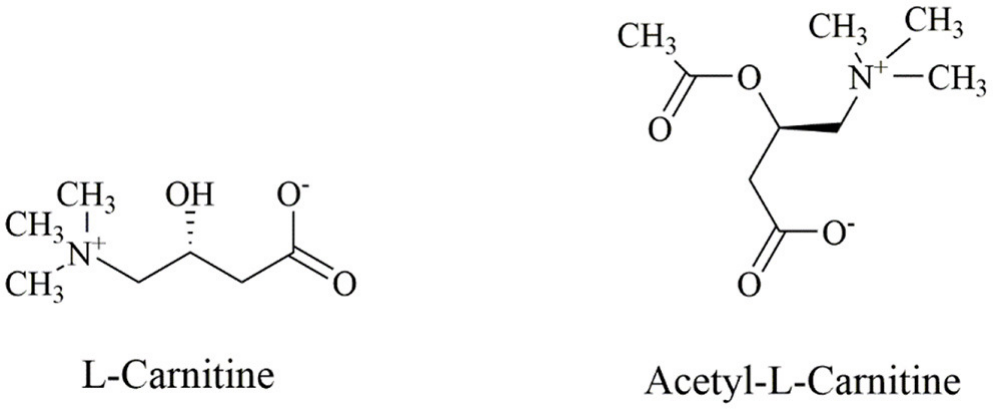
The structural formula of L-carnitine and Acetyl-L-carnitine.

Carnitine (3-hydroxy-4-N-trimethylaminobutyric acid) is a bioactive, water-soluble and low molecular weight amino acid that is usually considered as a dietary supplement (The structural formula of L-carnitine is shown in Figure 1). L-carnitine and D-carnitine isomers exist in mammals. However, only the L-carnitine isomers participate in the lipid metabolism (8). In humans, 75% of L-carnitine derives from diet, while the 25% endogenous part is synthesized from the essential amino acids, lysine, and methionine in the kidneys and liver (9). Furthermore, carnitine homeostasis is maintained *via* three pathways, namely dietary absorption, limited synthesis rate, and effective renal reabsorption (10).

**Figure 2:**
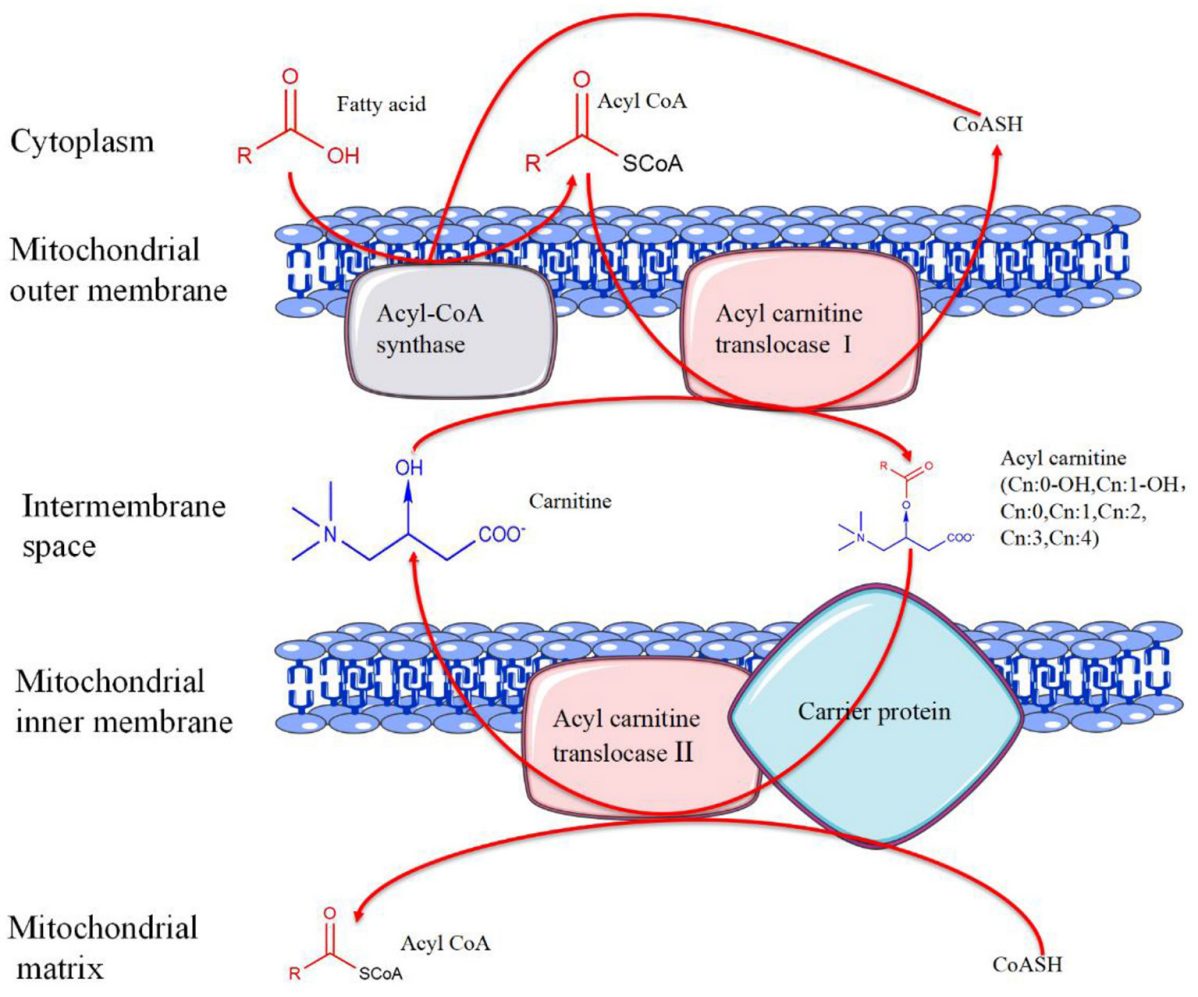
The metabolic process of carnitine. In the presence of coenzyme A (CoA)-SH, fatty acids are catalyzed by acyl-CoA synthase in the outer mitochondrial membrane to generate acyl-CoA. Activated acyl-CoA is transported into the mitochondria under the action of acylcarnitine translocase I with the help of carnitine and carrier protein, and acylcarnitine is produced at the same time. Subsequently, acylcarnitine releases carnitine under the action of acylcarnitine translocase II to produce acyl-CoA. Finally, acyl-CoA enters the mitochondrial matrix for further oxidation.

The hydroxyl group on carnitine can combine with organic acids or fatty acids to form different short-chain, medium-chain, and long-chain esters, namely ACs. ACs and free carnitine (FC) are the main forms of carnitine in human blood (11). ACs are the products of fatty acid β-oxidation, whose composition and concentration changes are also related to the occurrence and development of many diseases, such as congenital metabolic disorders (12), diabetes (13), atherosclerosis (14), tumors (15), and other diseases (The metabolic process of carnitine is summarized in Figure 2).

Carnitine, functioning in the mitochondrial fatty acid oxidation, also plays an important role in regulating the ratio of free coenzyme A (CoA) to acyl-CoA and in the removal of excessive acyl groups (11). In addition, exogenous carnitine derivatives have critical functions in the oxidation of glucose and can improve insulin resistance (16). Carnitine dysfunction may affect the lipid metabolism of mental disorders, suggesting that it may affect the occurrence and development of metabolic syndrome (17). Moreover, the occurrence of carnitine deficiency aggravates the symptoms in some diseases, such as encephalopathy (18), myopathy (19), and cardiomyopathy (20). The symptoms of these diseases are improved after carnitine supplementation (21). For cancer patients, the probability of carnitine deficiency has shown to be very high (22). A research showed that L-carnitine supplementation was safe and might have beneficial effects on fatigue, depressive symptoms and sleep quality in cancer patients (23).

## Acetyl-L-Carnitine and Physiological Function

Of the ACs in living organisms, LAC, as the most widely studied endogenous short-chain ACs, has the highest content of ACs and participates in cell anabolism and catabolism pathway (24). It naturally exists in the central nervous system and can travel through the blood-brain barrier (25). ACs have the functions of synthesizing lipids, changing and stabilizing membrane components, regulating gene expressions, improving mitochondrial function, increasing antioxidant activity, and enhancing cholinergic nerve transmission. It promotes acetyl-CoA to enter the mitochondria during fatty acid oxidation, produces acetylcholine, and stimulates the synthesis of protein and membrane phospholipids (26). Furthermore, Acetyl groups provided by LAC and fatty acid β-oxidation can be used to maintain the level of acetyl-CoA (27). LAC also binds with organic acids and free fatty acid fragments, allowing them to be excreted from the cells to prevent them from destroying the cell and tissue structures. In addition to its metabolic activities, LAC also has effects on cytoprotection, antioxidation, and anti-apoptosis in the nervous system (28).

## Carnitine and Depression

Increasing data seem to prove the hypothesis that carnitine levels are related to the progression of depression (Studies are summarized in Tables 1, 2). Zhang et al. (29) indicated that the average relative intensity of propionylcarnitine (C3:0), and butyrylcarnitine (C4:0) in plasma of overtired and depressed rats was lower than that of healthy control group. Another study found that most medium-chain and long-chain ACs increased in plasma of depressive rats (details are shown in Table 1). After treatments, most of ACs recovered to the normal level, except for the hydroxyoctadecadienoylcarnitine (C18:2-OH), octadecadienylcarnitine (C18:2), eicosadienoylcarnitine (C20:2). The increase of plasma long-chain ACs indicated that the fatty acid oxidation of depressed rats was incomplete, which may lead to a decrease in activities (30). Interestingly, researchers found that the plasma concentrations of carnitine and LAC were significantly higher in the CUMS-induced rat models than those in the control group, but there was no significant difference in the concentration of carnitine and LAC between the fluoxetine treatment group and the model group (31).

**Table 1:** Main information of pre-clinical researches studying the correlation between carnitine level and depression in animal.

**References**	**Samples**	**Subjects**	**Carnitine**	**Positive or negative**	**Main results**	**Depression scales or ways used**
Zhang et al. (29)	Plasma and urine	Control: *n* = 11; excess fatigue: *n* = 11; depression: *n* = 11	C3:0, C4:0	+	C3:0 and C4:0 in the overtired and depressed rats were lower than the control group.	Tail-hanging test
Chen et al. (30)	Plasma	Control: *n* = 12; depression: *n* = 12; Alliummacrostemon-treated depression: *n* = 11	Carnitine and ACs (C10:1, C12:0, C14:2, C14:1, C14:0, C16:2, C16:1, C16:0, C18:2, C18:1, C18:0, C20:2, C20:1, C20:0, C14:0-OH, C16:1-OH, C16:0-OH, C18:2-OH, C18:1-OH)	+	Most medium-chain and long-chain ACs (C10:1, C12:0, C14:2, C14:1, C14:0, C16:2, C16:1, C16:0, C18:2, C18:1, C18:0, C20:2, C20:1, C20:0, C14:0-OH, C16:1-OH, C16:0-OH, C18:2-OH, C18:1-OH) increased in the depression group. After treatment, most of the ACs decreased to the normal level, except for the C18:2-OH, C18:2, C20:2, but the three ACs also showed a significant tendency to the normal level.	Tail suspension test
Zhao et al. (31)	Plasma	Control: *n* = 8; CUMS: *n* = 8; fluoxetine treatment: *n* = 8	LAC and carnitine	–	The concentrations of LAC and carnitine in the model group were significantly higher than those in the control group, but there was no significant difference in the concentrations of carnitine and LAC between the fluoxetine treatment group and the model group.	Sucrose consumption

*Cm:n, Carnitine, in which “m” is the number of carbon atoms, “n” is the unsaturation. -OH represents hydroxylation, such as C18:1-OH = 3-hydroxy octadecenoyl carnitine*.

*ACs, acylcarnitines; CUMS, chronic unpredictable mild stress; LAC, acetyl-L-carnitine*.

**Table 2:** Detailed information of studies on carnitine and depression in human.

**References**	**Study type**	**Samples**	**Age (years)**	**Subjects**	**Carnitine**	**Positive or negative**	**Main results**	**Depression scales or ways used**
Nasca et al. (7)	Cross-sectional	Plasma	20–70	HC: *n* = 45; MDD: *n* = 71	LAC, free carnitine	+	The level of LAC in MDD patients was lower than healthy control, but the concentration of free carnitine had no significant difference.	HDRS-17 CTQ
Nie et al. (32)	Cross-sectional	Serum	Depression: 39.6 ± 18.27; HD: 42.53 ± 13.49	Depression: *n* = 100; HC: *n* = 100;	LC, LAC	+	LC and LAC were decreased in the depression group compared with healthy control.	Not mentioned
Liu et al. (33)	Cross-sectional	Plasma	Discovery set: HC: 43.98 ± 9.79, drug-naive MDD: 42.42 ± 11.01, validation set: HC: 33.67 ± 11.09, MDD: 36.04 ± 12.53	Discovery set: HC *n* = 59, drug-naive MDD: *n* = 60; Validation set: HC: *n* = 52, MDD: *n* = 75	ACs (C10:1, C10:2, C14:2, C14:3, C6:0, C8:0, C8:1, C10:0, C12:0, C12:1, C3:0)	–	Fifty-two identified metabolites showed significant differences between the two groups (including C10:1, C10:2, C14:2, C14:3, C6:0, C8:0, C8:1, C10:0, C12:0, C12:1, C3:0). The level of ACs decreased in MDD group compared with HC group.	DSM-IV HAMD
Mitro et al. (34)	Cross-sectional	Serum	>18	Pregnant woman with antenatal depression: *n* = 29; pregnant woman with non-antenatal depression: *n* = 71	C5:0 C5:1	+	C5:0 and C5:1 significantly decreased in the pregnant women with prenatal depression.	PHQ-9 DSM-IV
Ahmed et al. (35)	Cohort study	Plasma	CD+: 43.1 ± 13.9; ANX+: 36.8 ± 12.3; NVSM+: 36.6 ± 14.8	CD+: *n* = 31; ANX+: *n* = 44; NVSM+: *n* = 17	C0, C3:0, C4:0, C5-M-DC, C5:1, C5-OH, C8:0, C10:0, C12:0, C16:1, C18:1, C18:2	+	Free carnitine and acylcarnitines (C3:0, C4:0, C5-M-DC, C5:1) increased in the CD+ group. Free carnitine and acylcarnitines (C3:0 and C4:0) increased in the CD+ group and ANX+ group. Acylcarnitines (C8:0, C10:0, and C12:0) decreased in the CD+ group and NVSM+ group. Acylcarnitines (C5-OH, C16:1, C18:1, C18:2) decreased in all three MDD phenotypes.	HDRS-17
Nie et al. (36)	Cohort study	Serum	MDD: 36.92 ± 16.34; HC: 37.00 ± 15.22	MDD: *n* = 89; HC: *n* = 89	LC, LAC	+	LC and LAC were decreased in the MDD group. After treatment, LAC and LC were increased in the effective treatment group, but no significant change in the ineffective treatment group.	HDRS-24
Mahmoudiandehkordi et al. (37)	Cohort study	Plasma	40.17 ± 13.60	MDD: *n* = 136	Acylcarnitines C3:0, C4:0, C5:0, C8:0, C10:0, C12:0, C14:2, C16:0, C16:1, C18:0, C18:1 and C18:2	+	After 8 weeks of citalopram or escitalopram treatment in MDD patients, the levels of three short-chain acylcarnitines (C3:0, C4:0, and C5:0) increased, and the medium-chain and long-chain acylcarnitines (C8:0, C10:0, C12:0, C14:2, C16:0, C16:1, C18:0, C18:1, and C18:2) decreased.	HDRS-17
Moaddel et al. (38)	RCT	Plasma	18–65	Medication-free patients with treatment-resistant MDD: *n* = 29; HC: *n* = 25	C2:0	+	C2:0 decreased less in MDD after ketamine treatment than in the placebo group.	SCID-I BPRS
Akaishi et al. (39)	Cross-sectional	Serum	MS:36.4 ± 3.1 NMO:51.9 ± 13.6	Consecutive patients with multiple sclerosis: *n* = 75; patients with neuromyelitis optica: *n* = 39	Free carnitine AC Total carnitine	-	The low-levels of total carnitine, free carnitine and AC were identified in MS patients and NMO patients. The correlation coefficients between the serum carnitine levels and the three self-rated questionnaires were not significant between any pairs. Six MS patients and five NMO patients with a low-level of serum carnitine were administered L-carnitine. There was no significant improvement in any questionnaire after the treatment.	QIDS-SR PS ChFS
Jones et al. (40)	Cross-sectional	Plasma and urine	ME/CFS:M: 26–63, F: 21–84; Healthy: M: 20–66, F: 26–79; Depression: M: 34–71, F: 24–59; Rheumatoid arthritis: M: 43–67, F: 36–65	ME/CFS: M = 12, F = 19 Healthy: M = 12, F = 19 Depression: M = 6, F = 9 Rheumatoid arthritis: M = 5, F = 17	Total carnitine Free carnitine Esterified carnitine	–	No significant differences in urinary total, free or esterified (acyl) carnitine between UK patients with depression group and the control group.	SIPS
Sarris et al. (41)	RCT	Plasma	47.6 ± 13.2	SAMe: *n* = 18; escitalopram: *n* = 20; placebo: *n* = 16	Carnitine	–	There's no association between carnitine and response to SAMe or escitalopram.	SCID-I CGI-S HAMD-17
Yuan et al. (42)	Cross-sectional	Serum	HC: 57.00 ± 11.51; ESRD without depression: 50.18 ± 10.65; ESRD with depression: 50.82 ± 13.45	HC: *n* = 12; ESRD without depression: *n* = 17; ESRD with depression: *n* = 17	C3:0	+	Acylcarnitine C3:0 was decreased in the ESRD with depression than those without depression and C3:0 had a good ability to diagnose depression in the ESRD (AUC = 0.934).	HAMD HAMA
Fukami et al. (43)	Cross-sectional	Serum	HD: 63.4 ± 10.0 HC: 57.4 ± 7.1	Male HD: *n* = 26; healthy male: *n* = 15	Total carnitine Free carnitine AC	+	The level of free carnitine in male HD patients were significantly lower than those in age-matched healthy male subjects. The levels of AC and the ratio of AC to free carnitine in dialysis patients were significantly higher than those in age-matched healthy subjects.	AMS SDS
Tashiro et al. (44)	Cohort study	Serum	HD: 61.4 ± 10.5 HC:62.1 ± 7.9	HD male: *n* = 16; healthy male: *n* = 16	Total carnitine Free carnitine ACs (C3:0, C4:0, C5:0, C18:1-OH)	+	At baseline, the levels of total carnitine and free carnitine in dialysis patients were lower than those in healthy controls, but the levels of ACs in dialysis patients were higher than that in healthy controls. The levels of free carnitine and C3:0 were significantly lower and C4:0, C5:0, C18:1-OH were higher in HD patients than in healthy subjects. L-carnitine supplementation significantly increased serum levels of free and other ACs.	SDS
Cassol et al. (45)	Cross-sectional	Plasma	HIV-positive test cohort: 35–60, HIV-negative cohort: 35–62	HIV-positive: *n* = 68; HIV-negative: *n* = 36	C3:0, isoC4:0, isoC5:0, 2-methyl C4:0	+	ACs (C3:0, iso C4:0, iso C5:0, 2-methyl C4:0) were decreased to distinguish depressed subjects from controls in HIV-positive and HIV-negative cohorts.	BDI CES-D
Rezaee et al. (46)	Cross-sectional	Serum	35.34 ± 8.02	AIDs: *n* = 100	Carnitine	–	A non-statistically significant negative correlation between depression scores and total levels of serum carnitine was detected. No significant correlation was found between dietary carnitine intake and serum carnitine levels.	BDI

*Cm:n, Carnitine, in which “m” is the number of carbon atoms, “n” is the unsaturation. -OH represents hydroxylation, such as C18:1-OH, 3-hydroxy octadecenoyl carnitine*.

*AC, acylcarnitine; AIDs, Acquired Immune Deficiency Syndrome; AMS, aging male symptoms scale; ANX+, anxious depression; BDI, Beck Depression Inventory; BPRS, Brief Psychiatric Rating Scale; CD+, core depression; CES-D, the Center for Epidemiological Studies Depression Scale; CFS, chronic fatigue syndrome; CGI-S, Clinical Global Improvement-severity; ChFS, Chalder fatigue scale; CTQ, Childhood Trauma Questionnaire; DSM-IV, the fourth Diagnostic and Statistical Manual of Mental Disorders criteria; ESRD, end-stage renal disease; F, female; HAMD, The Hamilton depression scale; HAMD-17, 17-item Hamilton-D Scale; HC, healthy control; HD, hemodialysis; HDRS-17, 17-item Hamilton Depression-Rating Scale; HIV, human immunodeficiency virus; LAC, acetyl-L-carnitine; M, male; MDD, major depressive disorder; ME, myalgic encephalomyelitis; MS, multiple sclerosis; NMO, neuromyelitis optica; NVSM+, Neurovegetative symptoms of melancholia; PHQ-9, Patient Health Questionnaire 9; PS, performance status; QIDS-SR, self-reported quick inventory of depressive symptomatology; RCT, randomized controlled trial; SAMe, S-adenosyl methionine; SCID-I, the Structured Clinical Interview for Axis I DSM-IV Disorders-Patient Version; SDS, self-rating depression scale; SIPS, Sickness Impact Profile Score*.

Furthermore, the relationship of carnitine and depression has also been explored in different clinical studies. Nasca et al. (7) found that LAC levels in patients with MDD decreased compared with healthy controls, but there was no significant difference in free carnitine. However, another study found that free carnitine was decreased in depression group (32). Moreover, LAC declined more sharply in patients with a history of refractory depression. A history of childhood trauma and emotional neglect (only in women) can be used as the factors to predict the concentration of LAC in refractory depression patients (7). One study explored the differences in plasma metabonomics between MDD patients and healthy people, in which 52 identified metabolites showed differences between the two groups, including the levels of decenoylcarnitine (C10:1), decadienylcarnitine (C10:2), tetradecadienylcarnitine (C14:2), tetradecatrienecarnitine (C14:3), hexanoylcarnitine (C6:0), octanoylcarnitine (C8:0), decanoylcarnitine (C10:0), dodecanoylcarnitine (C12:0), and C3:0. The concentration of above carnitines was negatively correlated with the score of the Hamilton scale (33). Mitro et al. (34) reported that C5:0 and C5:1 were associated with lower odds of antepartum depression whereas C4:0 and C5:0 were with continuous Patient Health Questionnare-9 (PHQ-9) score. Ahmed et al. (35) found that acylcarnitine metabolism can be used to distinguish MDD phenotypes. Free carnitine and short-chain acylcarnitines (C3:0, C4:0, C5-M-DC, C5:1) increased in the core depression (CD+) group. Free carnitine and acylcarnitines (C3:0 and C4:0) increased in the CD+ group and anxious depression (ANX+) group. Acylcarnitines (C8:0, C10:0, and C12:0) decreased in the CD+ group and neurovegetative symptoms of melancholia (NVSM+) group. Acylcarnitines (C5-OH, C16:1, C18:1, C18:2) decreased in all three MDD phenotypes. Furthermore, the acylcarnitine levels of MDD patients changed significantly after treatment. For MDD patients after 2 weeks treatment, LC and LAC levels in the effective treatment group increased significantly, but no significant changes were seen in the ineffective treatment group (36). After 8 weeks of citalopram or escitalopram treatment in MDD patients, the levels of three short-chain acylcarnitines (C3:0, C4:0, and C5:0) increased, and the medium-chain and long-chain acylcarnitines (C8:0, C10:0, C12:0, C14:2, C16:0, C16:1, C18:0, C18:1, and C18:2) decreased (37). In a randomized controlled trial (RCT), Moaddel et al. (38) found that LAC decreased less after ketamine treatment in the MDD group than it did in the placebo group. It demonstrated that low molecular weight compounds in the body can be used as biomarkers for diagnosing and prognosis evaluation of depression. However, some studies have found that the level of carnitine is not significantly correlated with depression. Some patients with low serum carnitine level had no significant improvement after oral administration of L-carnitine (39). Levels of carnitine (total carnitine, free carnitine, ACs) in urine did not differ significantly between healthy controls and patients with depression (40). Jerome et al. (41) found no association between baseline carnitine level and response to S-adenosyl methionine (SAMe) or escitalopram treatments.

Many comorbidities of depression are associated with changes of carnitine levels. Depression is common in hemodialysis (HD) patients (47) which may reduce drug compliance and have a negative impact on the quality of life in HD patients (48). Studies showed that the levels of total carnitine, free carnitine, L-carnitine and C3:0 in dialysis patients were lower than those in healthy controls, while those of C4:0, isoamyl C4:0 and C18:1-OH were higher. Another study also found that acylcarnitine C3:0 was decreased in the end-stage renal disease (ESRD) with depression than those without depression and C3:0 had a good ability to diagnose depression in the ESRD (AUC = 0.934) (42). As long-chain ACs are excreted and metabolized in the kidney, it might be the reason for the increase of serum AC levels in male uremic HD patients. Renal diseases generally lead to L-carnitine homeostasis imbalance as long-term hemodialysis leads to an increase in the ratio of L-AC to free L-carnitine. Such changes interfere with the oxidation of fatty acids and the removal of unwanted short-chain acyl groups from tissues (43, 49). Supplementation of carnitine helps in decreasing self-rating depression scale (SDS) score and improving depressive symptoms in the dialysis group. The state of depression was related to the level of carnitine (44).

Carnitine also plays an important role in other diseases associated with depression, like multiple sclerosis (MS), neuromyelitis optica (NMO), and HIV infection. Decreased levels of total carnitine, free carnitine, and ACs were found in MS and NMO patients. As the most debilitating symptoms, depressive state, and chronic fatigue occur in a majority of patients with MS and NMO (39, 50). Moreover, carnitine deficiency, either from reduced intake or impaired endogenous synthesis, is considered to be the cause of fatigue, which can be treated with oral LAC supplementation (51, 52). Interestingly, there was no difference in carnitine levels between MS patients with or without fatigue. One study revealed that mitochondrial AC deficiency and fatty acid metabolism disorders were not related to fatigue in patients with MS (53). Another study also found no significant correlation between serum carnitine level and scores of three self-evaluation scales in NMO and MS. Determination of serum L-carnitine levels and the benefits of oral administration of L-carnitine remain unclear (39). Depression is also a common complication among patients with HIV infection for their poor treatment outcomes and high mortality (54). Cassol et al. (45) found decreased monoamine metabolites (phenylacetate, 4-hydroxyphenylacetate) and ACs (C3:0, iso C4:0, iso C5:0, 2-methyl C4:0) in plasma distinguished depressed subjects from controls in HIV-positive and HIV-negative cohorts, and these alterations were correlated with the severity of depression. However, a study showed that no significant correlation was found between depression and serum carnitine level as well as dietary carnitine intake (46). In view of the conflicting results presented above, future research should be conducted in larger samples.

Various factors can affect the level of carnitine in vivo. Previous studies have found that age was a factor in determining carnitine level (55, 56). In aged people, the entry of carnitine into cells is negatively affected, thus reducing the metabolism of LAC. Moreover, age has found to be positively correlated with higher AC/FC ratio. Elderly patients with psychosis may suffer secondary carnitine deficiency as higher serum AC/FC ratio is an indicator of secondary carnitine deficiency (21). Besides, the renal reabsorption of LAC may also be impaired, leading to an increase in carnitine level and a decrease in LAC level (57). With regard to gender, there was a significant negative correlation between estrogen level and serum free carnitine in females (58). Therefore, when designing the carnitine determination experiment, in order to avoid the interference of baseline confounding factors, we must pay special attention to the consistency of age, gender, and renal function between the groups, so that the final determination results are comparable.

## Carnitine in Bipolar Disorder

Bipolar disorder (BD) is a chronic mental illness characterized by severe bipolar changes, ranging from extreme excitement or mania to severe depression. No effective biomarkers have been found for BD, making accurate diagnosis difficult in clinical practice (59). Abnormal mitochondrial function has been found to be present in patients with BD (60). Therefore, several readily-made dietary supplements, including LAC, have been tested as potential treatments for BD with mitochondrial regulatory functions (61, 62). Although LAC supplement is a simple and cost-effective adjuvant therapy used for the treatment of most neurological diseases, its therapeutic effect on BD is controversial (63). Dosage and duration of LAC and α-lipoic acid (ALA) have shown no antidepressant effect on patients with BD, which also had insignificant enhancement of mitochondrial function in patients (62, 64).

A cross-sectional study found that outpatients had higher total carnitine levels in serum than acutely hospitalized schizophrenia and BD patients, but there was no significant difference in total and free carnitine levels of patients with schizophrenia or BD. Serum carnitine is therefore not a reliable marker to distinguish schizophrenia from BD (65). Valproic acid (VPA) is commonly used in several neurological and psychiatric indications. It was found that the dosage of VPA was negatively correlated with the levels of total carnitine and free carnitine, suggesting that sodium valproate had a negative effect on the bioavailability and metabolism of carnitine (55, 66). Another study indicated that the concentration of C2:0 in BD with hyperammonemia and treated with VPA were significantly lower than epilepsy group under phenytoin treatment. Carnitine had a downward trend. This result showed that the level of human blood ammonia was related to the concentrations of carnitine and LAC (56) (Studies are summarized in Table 3).

**Table 3:** Main information of clinical researches studying the correlation between carnitine level and bipolar disorder.

**References**	**Study type**	**Samples**	**Age (years)**	**Subjects**	**Carnitine**	**Positive or negative**	**Main results**	**Bipolar disorder scales or ways used**
Maldonado et al. (56)	Cross-sectional	Dried blood spot specimens	Group A: range 18–55; Group B: range 18–55; Group C: range 65–85	A: epileptic patients treated with phenytoin alone: *n* = 31; B: bipolar disorder patients treated with valproic acid: *n* = 28; C: aged people: *n* = 41	Carnitine and C2:0	–	The concentration of C2:0 in BD with hyperammonemia and treated with VPA were significantly lower than epilepsy group under phenytoin treatment. Carnitine has a downward trend.	Not mentioned
Cuturic et al. (65)	Cross-sectional	Serum	Impatient mean age: 48.7 ± 12.2 Outpatients mean age: 49.8 ± 11.3	Schizophrenia: *n* = 30; bipolar disorder type 1: *n* = 30	Total carnitine Free carnitine	+	Total carnitine in outpatients was higher than psychiatric patients in emergency department. Carnitine in patients who met the criteria of metabolic syndrome was higher than patients without metabolic syndrome. No statistical difference in carnitine levels between African Americans and Caucasians patients diagnosed with schizophrenia and bipolar disorder.	Not mentioned

## Carnitine in Schizophrenia

Carnitine has potential diagnostic and therapeutic effects on schizophrenia (67, 68). Tasic et al. (69) found that C5:0 was present only in schizophrenics, but not in BD and healthy people, suggesting that carnitine was a biomarker to identify schizophrenia. For patients with schizophrenia and metabolic syndrome (MetS), 8 plasma ACs [C3:0, C4:0, C5:0, hexenoylcarnitine (C6:1), C10:1, C10:2, hydroxytetradecadienylcarnitine (C14:2-OH), hydroxyhexadecadienylcarnitine (C16:2-OH)] were significantly higher than those in schizophrenics without MetS (70). Moreover, concentrations of ACs [hydroxybutyrylcarnitine (C4:0-OH) and hexadecenoylcarnitine (C16:1)] in schizophrenia patients were significantly higher than those in healthy controls, whereas those of C3:0, C8:0, C10:0, C10:1, C10:2, C12:0, hydroxytetradecenoylcarnitine (C14:1-OH), C14:2 and C14:2-OH were lower. After treatment, the levels of acetylcarnitine (C2:0), C8:0, C10:0, C10:1, C10:2, C12:0, C12:1, C14:1, C14:1-OH, C14:2, C14:2-OH, C16:2, C16:2-OH, and C18:0 decreased compared to the controls (71). A longitudinal study demonstrated that the levels of C18:0, L-C16:0, C18:2, and LAC decreased in the first-episode and recurrent patients after 8 weeks treatment (72). However, LAC combined with clozapine was found to not significantly improve the treatment effect of schizophrenic patients who were tolerant to clozapine (73) (Studies are summarized in Table 4).

**Table 4:** Main information of clinical researches studying the correlation between carnitine level and schizophrenia.

**References**	**Study type**	**Samples**	**Age (years)**	**Subjects**	**Carnitine**	**Positive or negative**	**Main results**	**Schizophrenia scales or ways used**
Tasic et al. (69)	Cross-sectional	Serum	18–65	Schizophrenia: *n* = 54; euthymic outpatients with BD type 1: *n* = 68; healthy: *n* = 60	IsoC5:0	+	Iso C5:0 exists only in schizophrenics, but not in bipolar disorder and normal subjects.	SCID-I PANSS GAF YMRS HDRS-17
Cao et al. (70)	Cross-sectional	Plasma	Schizophrenia with MetS: 41.30 ± 9.78; Schizophrenia without MetS: 35.57 ± 10.55	Schizophrenia with MetS: *n* = 46; schizophrenia without MetS: *n* =1 23	ACs (C3:0, C4:0, C5:0, C6:1, C10:1, C10:2, C14:2-OH, C16:2-OH)	+	Eight plasma ACs (C3:0, C4:0, C5:0, C6:1, C10:1, C10:2, C14:2-OH, C16:2-OH) in patients with schizophrenia and MetS were significantly higher than those in schizophrenics without MetS.	DSM-IV
Cao et al. (71)	Cohort study	Plasma	Schizophrenics: 37.31 ± 10.85; healthy controls: 39.44 ± 9.36	Schizophrenia: *n* = 225; healthy controls: *n* = 175	ACs (C2:0, C3:0, C4-OH, C8:0, C10:0, C10:1, C10:2, C12:0, C12:1, C14:1, C14:1-OH, C14:2, C14:2-OH, C16:1, C16:2, C16:2-OH, and C18:0)	+	Concentrations of ACs (C4-OH and C16:1) in schizophrenia patients were significantly higher than that in healthy controls, while C3:0, C8:0, C10:0, C10:1, C10:2, C12:0, C14:1-OH, C14:2 and C14:2-OH were lower than healthy controls. After treatment, the levels of C2:0, C8:0, C10:0, C10:1, C10:2, C12:0, C12:1, C14:1, C14:1-OH, C14:2, C14:2-OH, C16:2, C16:2-OH, and C18:0 decreased compared to the control.	PANSS CRF
Cao et al. (72)	Cohort study	Serum	28.91 ± 6.21	First-episode and drug-naïve: *n* = 29; recurrent or chronic schizophrenia: *n* = 93	C18:0 L-C16:0 C18:2 LAC	+	C18:0, L-C16:0, C18:2, and LAC were decreased after treatment.	DSM-IV PANSS CRF

*The age was expressed as MEAN ± SD. Carnitine is expressed as Cm: n, “m” is the number of carbon atoms, “n” is the unsaturation. If carnitine is hydroxylated, you can could put a hydroxide in the end, like 3-hydroxy octadecenoyl carnitine (C18:1-OH)*.

*AC, acylcarnitine; CRF, Case Report Form; DSM-IV, Diagnostic and Statistical Manual of Mental Disorders; GAF, The Global Assessment of Functioning Scale; HDRS-17, the Hamilton Depression Rating Scale-17; LAC, acetyl-L-carnitine; MetS, metabolic syndrome; PANSS, The Positive and Negative Syndrome Scale; SCID-I, The Structured Clinical Interview for Axis I; YMRS, Young Mania Rating Scale*.

## The Potential Antidepressant Mechanisms of Acetyl-L-Carnitine

**Figure 3:**
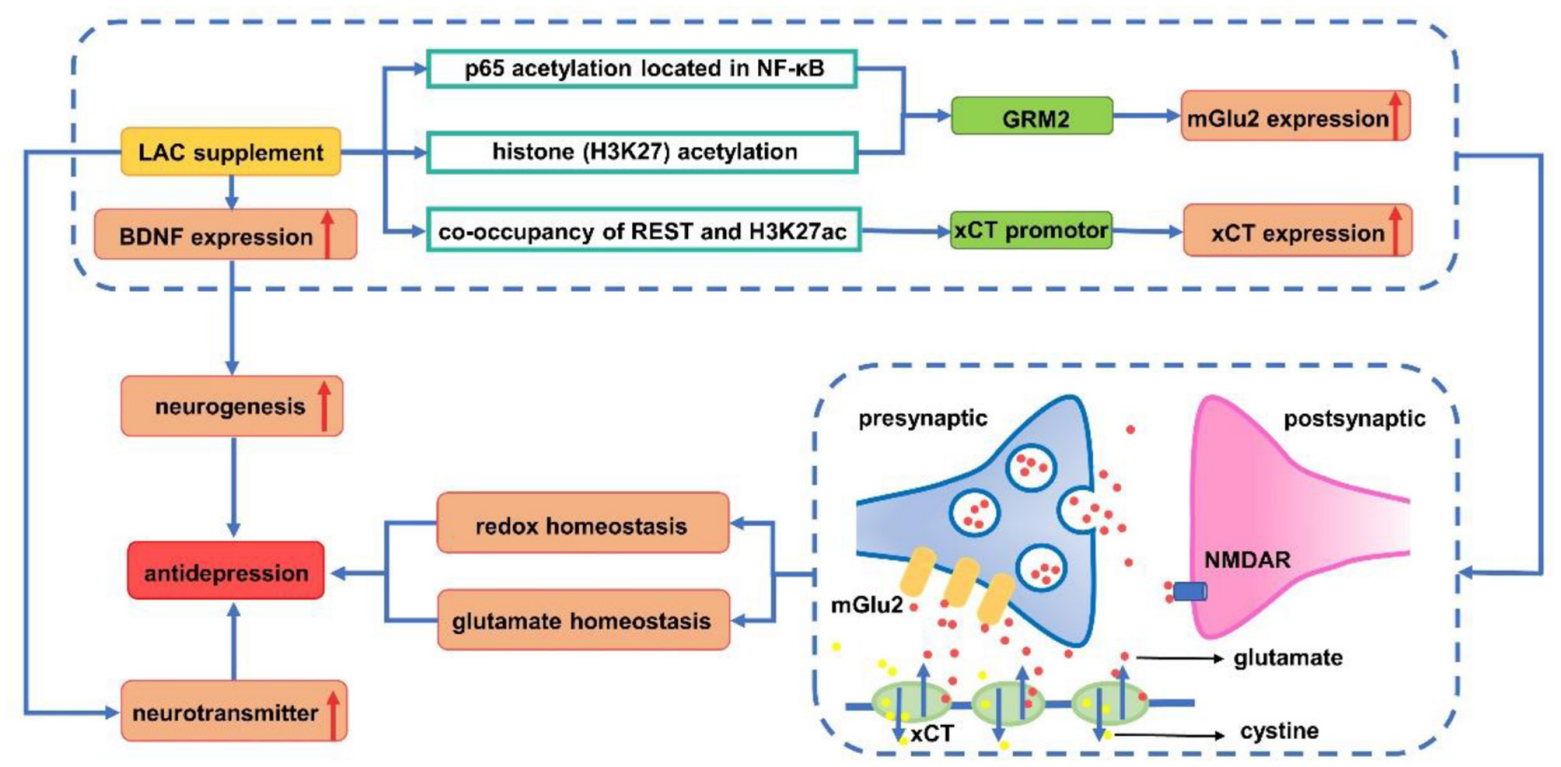
Antidepressant mechanism of acetyl-L-carnitine. LAC supplement increases the expression of mGlu2 (*via* up-regulating transcription of GRM2) by acetylating p65 on NF-κB and H3K27. REST (repressor element 1 silencing transcription factor) and H3K27ac are enriched in the xCT promoter, promoting the expression of xCT. This in turn causes the release of glutamate out of the cell, which activates the mGlu2 receptor, and reduces the spillover of glutamate between synapses to enhance glutamate homeostasis. Meanwhile, xCT transports cystine into cells for the synthesis of glutathione to avoid oxidative stress damage. Upregulation of BDNF expression induced by LAC increases neurogenesis to exert antidepressant effects. Increase of monoamine neurotransmitters (such as 5-HT, DA, and NE) induced by LAC has demonstrated its antidepressant effect from the pathogenesis of depression. BDNF, brain derived neurotrophic factor; DA, dopamine; GRM2, glutamate receptor metabotropic 2; H3K27ac, acetylated H3K27; 5-HT, serotonin; LAC, acetyl-L-carnitine; mGlu2, metabotropic glutamate 2; NE, norepinephrine; NMDAR, N-methyl-D-aspartic acid receptor; REST, repressor element silencing transcription factor; xCT: Xc-cystine/glutamate antiporter.

About 2/3 of patients with severe depression are unable to achieve complete remission of symptoms with antidepressants currently available (74, 75). In recent years, there have been a few new and unique antidepressants (76). It has been found that the odds of developing any adverse events are highest for tricyclic antidepressants and serotonin noradrenaline reuptake inhibitors. However, since LAC is an endogenous molecule, it is safer compared with other medicine (77, 78). Unlike some currently used antidepressants that take effect within 2–3 weeks, LAC can take effect within 2–3 days (79). LAC can be used in patients with MDD as a cognitive enhancer (80) and carnitine supplementation is beneficial to depression. LAC supplementation, with the same effect as antidepressants, can significantly reduce depressive symptoms compared with placebo/no intervention (81). Therefore, it is expected to become the next generation of new antidepressants with fewer side effects (79). Previous studies have proposed a variety of mechanisms of LAC in the treatment of depression. Most studied potential mechanisms include neuroplasticity effect, oxidative stress and neurotransmitter regulation. The antidepressant mechanisms of LAC are summarized in Figure 3.

### Neuroplasticity Effect

The adult brain can adapt to internal and external stressors through structural plasticity, and failed resilience is likely to cause depression and anxiety. Behaviorally, the LAC treatment during the last part of chronic restraint stress (CRS) enhanced resilience and reversed the effect of CRS, which is shown by increased social interaction and reduced passive behavior in a forced swim test (82).

Disorders of metabotropic glutamate (mGlu) receptors and neurotrophins have significant negative effects on neuroplasticity, leading to depression, but LAC regulate brain type 2 metabotropic glutamate (mGlu2) receptors and N-methyl-D-aspartic acid (NMDA) receptors to induce antidepressant effect (83–85). In detail, it up-regulated level of acetylated H3K27 induced by LAC that binds to the glutamate receptor metabotropic 2 (GRM2) promoter and acetylation of the NF-κB p65 subunit, which enhanced the transcription of the GRM2 gene encoding mGlu2 receptor in the hippocampus and prefrontal cortex (7, 86). While improving central energy regulation and playing a rapid antidepressant-like effect, LAC corrected systemic hyperinsulinemia and hyperglycemia in responder Flinders Sensitive Line (rFSL). As LAC can regulate metabolic factors and reduce glutamate spillover, it can quickly improve depression and also be considered for the treatment of insulin resistance in patients with depression (85).

Neurotrophic factors are associated with the pathogenesis of depression (87, 88). The decreased concentrations of BDNF (89), glial cell line-derived neurotrophic factor (GDNF), Artemin (ARTN) (90), nerve growth factor (NGF) (91), and vascular endothelial growth factor (VEGF) (92) are a good repetitive finding in depression. Supplementation of LAC can induce neuroprotective and neurotrophic effects of the peripheral nervous system by increasing the level of brain-derived neurotrophic factor (BDNF), especially in the hippocampus and prefrontal cortex (93–95). Meanwhile, it could promote the growth of dendrites and increase the number of intersections in the medial amygdala (82). In addition, it has a positive effect on the differentiation of neural progenitor cells in hippocampus, proliferation, and long-term survival of neural progenitor cells (84, 96). Studies have shown that LAC can reverse the decrease in NGF caused by pressure exposure in the central nervous system (97), and normalize the levels of GDNF and ARTN in the peripheral and central nervous systems (98), but there is still a lack of relevant research on the effect of LAC on VEGF in mental illness.

### Oxidative Stress

LAC promoted the removal of oxidation products and provided acetyl groups to regulate the expression of neuro-nutrients and glutamate genes (25, 99). xCT (Xc^−^ cystine/glutamate antiporter) is an important component of antioxidant activity *in vivo*, which is associated with glutathione synthesis (100). Pharmacologically, modulating histone acetylation with LAC rapidly increased xCT and activated a network with mGlu2 receptors to prime enhanced glutamate homeostasis that promotes both pro-resilient and antidepressant-like responses (101).

### Neurotransmitter Regulation

The neurotransmitters associated with depression mainly include monoamine, 5-methyl-hydroxytryptamine, and dopamine (DA) (102). Long-term LAC supplementation reduces glucose metabolism to lactic acid, resulting in an increment in energy metabolites and changes in norepinephrine (NE), DA and serotonin (5-HT) in the brain of mice (96, 103). WAY-100635, a 5-HT_1A_ antagonist, could significantly inhibit the antidepressant effects of acetyl-L-carnitine. It is suggested that 5-HT_1A_ may be the target of LAC treatment. Effects of long-term administration of LAC on rats increased dopamine production in the marginal division of the rat cortex and protective effects on acute stress exposure (96). Moreover, Up-regulation of γ-aminobutyric acid (GABA) levels in the brain is considered to be related to the pathological mechanism of depression (104). Long-term LAC can significantly reduce the GABA level in the hippocampus of mice, but the GABA level in the cortex did not change significantly (103).

## Discussion

Although many antidepressants with different mechanisms are being used, a number of patients have not achieved appropriate therapeutic effects. The monoamine hypothesis of depression has been the main pathophysiology and drug therapy target of depression over the past few decades, but the current monoamine theory has serious limitations (105). Therefore, new antidepressants with different mechanisms are needed to enable clinicians to diversify the treatment options for MDD. Perhaps LAC may be the next potentially effective and tolerable treatment with new mechanisms for patients with depression, especially for elderly and female patients, as well as patients with concomitant diseases who are vulnerable to the adverse effects of antidepressants. However, compared with other antidepressants, its hypothetical benefits must be thoroughly studied. More large-scale clinical trials are needed to determine whether LAC as a single drug or enhancer is effective and clinically beneficial for the treatment of depression.

The relationship of multiple ACs *in vivo* and psychiatric disorders such as depression has yet to be further investigated. Most of the current studies on depression have found that the levels of free carnitine and several short-chain acylcarnitines decreased in patients with depression. The changes of medium- and long-chain acylcarnitines are not well-studied but their metabolism may have to do with comorbiditic diseases (43, 49). One Study have found that iso C5:0 exists only in patients with schizophrenia, not in those with bipolar disorder and normal subjects (69), but it still exists in depressive patients (45). Therefore, iso C5:0 may be an important biomarker to distinguish between depression, schizophrenia and BD. Interestingly, it is found that C8:0, C10:0, C10:1, C12:0, and C14:2 have opposite changes in depression and schizophrenia compared with healthy controls (30, 37, 71). The differences in the metabolism of these ACs may potentially become important biomarkers for distinguishing depression and schizophrenia, but these possibilities are yet to be explored in further research (Table 5).

**Table 5:** Changes of different acylcarnitine in mental diseases compared with healthy controls.

**Biochemicalname**	**Abbreviation**	**Change trend in depression**	**Change trend in schizophrenia**
Acetylcarnitine	C2:0	↓	?
Propionylcarnitine	C3:0	↓	↓
Butyrylcarnitine	C4:0	↓	?
Hydroxybutyrylcarnitine	C4:0-OH	?	↑
Valerylcarnitine	C5:0	↓	?
Tiglylcarnitine	C5:1	↓	?
Hexanoylcarnitine	C6:0	?	?
Hexenoylcarnitine	C6:1	?	?
Hydroxyhexanoylcarnitine	C6:0-OH	?	?
Pimelylcarnitine	C7-DC	?	↑
Octanoylcarnitine	C8:0	**↑**	**↓**
Octenoylcarnitine	C8:1	?	?
Decanoylcarnitine	C10:0	**↑**	**↓**
Decenoylcarnitine	C10:1	**↑**	**↓**
Decadienylcarnitine	C10:2	?	↓
Dodecanoylcarnitine	C12:0	**↑**	**↓**
Dodecenoylcarnitine	C12:1	?	?
Tetradecanoylcarnitine	C14:0	↑	?
Tetradecenoylcarnitine	C14:1	↑	?
Tetradecadienylcarnitine	C14:2	**↑**	**↓**
Tetradecatrienecarnitine	C14:3	?	?
Hydroxytetradecanoylcarnitine	C14:0-OH	↑	?
Hydroxytetradecenoylcarnitine	C14:1-OH	?	↓
Hydroxytetradecadienylcarnitine	C14:2-OH	?	↓
Hexadecanoylcarnitine	C16:0	↑	?
Hexadecenoylcarnitine	C16:1	↑	↑
Hexadecadienylcarnitine	C16:2	↑	?
Hydroxyhexadecanoylcarnitine	C16:0-OH	↑	?
Hydroxyhexadecenoylcarnitine	C16:1-OH	↑	?
Hydroxyhexadecadienylcarnitine	C16:2-OH	?	?
Octadecanoylcarnitine	C18:0	↑	?
Octadecenoylcarnitine	C18:1	↑	?
Octadecadienylcarnitine	C18:2	↑	?
Hydroxyoctadecenoylcarnitine	C18:1-OH	↑	?
Hydroxyoctadecadienoylcarnitine	C18:2-OH	↑	?
Eicosylcarnitine	C20:0	↑	?
Eicosaenoylcarnitine	C20:1	↑	?
Eicosadienoylcarnitine	C20:2	↑	?

Meanwhile, the determination of carnitine concentrations in serum or plasma did not seem to be significantly different. The comprehensive screening and evaluation of carnitine changes in patients with depression is of great significance to studying the development of depression in depth. It is believed that ACs, especially LAC, might become potential biomarkers for the diagnosis of depression and effective drugs for their treatment of depression in the future given their important clinical value. Comprehensive studies on the relationships of depression and carnitine metabolism can lend support to the diagnosis and treatment of other psychiatric diseases such as BD and schizophrenia, so as to further study the similarities and differences of carnitine metabolism in psychiatric diseases.

## Author Contributions

TL and KD wrote the manuscript. YX collected the relevant literature and organized it. RuY and RoY checked the content of the manuscript. ZG and MT reviewed and amended the manuscript. All authors read and approved the final manuscript.

## Funding

The work was supported by National Natural Science Foundation of China (No. 81803233), the Natural Science Foundation of Hunan Province (No. 2018JJ3834), the Key Research Project of Ningxia Hui Autonomous Region in 2021 (Major Project) (2021BEG01001), Science Foundation of Xiangya Hospital for Young Scholar (No. 2017Q13), and China Postdoctoral Science Foundation (No. 2021M693561).

## Conflict of Interest

The authors declare that the research was conducted in the absence of any commercial or financial relationships that could be construed as a potential conflict of interest.

## Publisher's Note

All claims expressed in this article are solely those of the authors and do not necessarily represent those of their affiliated organizations, or those of the publisher, the editors and the reviewers. Any product that may be evaluated in this article, or claim that may be made by its manufacturer, is not guaranteed or endorsed by the publisher.
